# Supercapacitive paper based microbial fuel cell: High current/power production within a low cost design

**DOI:** 10.1016/j.biteb.2019.100297

**Published:** 2019-09

**Authors:** Carlo Santoro, Jonathan Winfield, Pavlina Theodosiou, Ioannis Ieropoulos

**Affiliations:** Bristol BioEnergy Centre, Bristol Robotics Laboratory, T-Block, UWE, Coldharbour Lane, Bristol BS16 1QY, UK

**Keywords:** Microbial fuel cell, Urine, Supercapacitive mode, Galvanostatic discharges, Low-cost system

## Abstract

Microbial fuel cells (MFCs) with paper separators and liquid containing elements were investigated in supercapacitive mode. MFCs (15 mL) in a supercapacitive configuration, consisted of plain wrapped carbon veil anode (negative) and conductive latex cathode (positive). The internal supercapacitor is discharged galvanostatically and is self-recharged as red-ox reactions occur on both electrodes. MFCs were discharged at different current pulses varying from 1 mA to 7 mA. The MFCs had an equivalent series resistance of 41.2 ± 3.5 Ω caused mainly by the cathode. A maximum power of 1.380 ± 0.083 mW (0.092 ± 0.006 mW mL^−1^) was measured. Durability tests were conducted over 24 h collecting 1000 discharge cycles (0.5 s) and self-recharges (85 s) at a current of 1 mA. Over time the anode potential dropped causing a decline in performance perhaps due to evaporation of liquid from the pyramidal structure. Resistance and apparent capacitance measured during the durability test remained approximately constant during the cycles.

## Highlights

• Supercapacitor paper-based MFCs are reported for the first time.
• Galvanostatic discharges at different current pulses (1 mA to 7 mA) were analysed.
• Equivalent series resistance (ESR) and apparent capacitance (C) were identified.
• Maximum power achieved was 1.380 ± 0.083 mW (0.092 ± 0.006 mW mL^−1^).
• The system stability was investigated through 1000 discharge/self-recharge cycles.

## Graphical abstract

**Figure:**
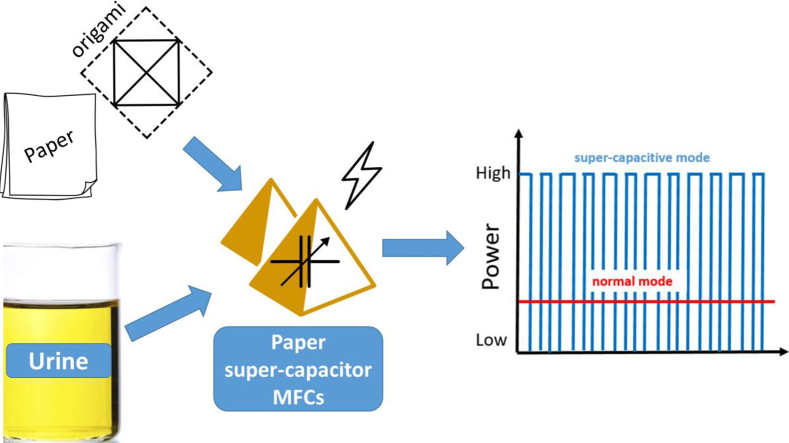

## 1 Introduction

Society is facing significant environmental challenges that are damaging the global climate, rendering our environment uncertain for future generations. Novel sustainable technologies need to be incorporated into current processes to deal with high energy demand, water scarcity and waste management, all of which impact on health and quality of life. Bioelectrochemical systems (BES) are a group of technologies that can help address these issues (Bennetto, 1990; Logan et al., 2006). All BES technologies employ electroactive bacteria to oxidise organic matter in a liquid (e.g. wastewater) (Bennetto, 1990; Logan et al., 2006). Among BESs, microbial fuel cells (MFCs) are of particular interest because the degradation of organics results in the simultaneous production of electricity (Potter, 1911). The microbes inhabit the anode chamber where organic compounds are oxidised and this is coupled with the reduction of an oxidant at the cathode electrode. The natural cathodic oxidant of choice is oxygen because of its high redox potential and atmospheric abundance (Bennetto, 1990).

Individual MFCs produce relatively low power and therefore improvements, for example, to the electrode materials, are needed to enhance the electrochemical output (Guo et al., 2015). For the anode, the focus needs to be on increasing the biotic-abiotic interface for bacterial attachment, and on improving the material conductivity by adding functional groups or highly conductive carbon (Guo et al., 2015; Wei et al., 2011). Generally anode electrodes are composed of conductive carbonaceous materials with high surface area and possess characteristics that contribute to durability and stability over time (Wei et al., 2011).

In terms of the cathode, the oxygen reduction reaction (ORR) is sluggish in neutral media but can be accelerated by decorating the cathodes with inorganic catalysts (Kodali et al., 2017, Kodali et al., 2018). While platinum and platinum group metals (PGM) were once commonplace as cathode catalysts (Wang et al., 2014), low cost, metal-free carbonaceous materials are currently the catalysts of choice (ElMekawy et al., 2017; Santoro et al., 2017) as they can be decorated with transition metals/materials (e.g. Ni, Fe, Mn or Co) (Rojas-Carbonell et al., 2017, Rojas-Carbonell et al., 2018). PGM-free catalysts have been reported to be robust and efficient for catalysing oxygen in neutral media (Costa de Oliveira et al., 2017; Gajda et al., 2018; Santoro et al., 2018a).

The design of the MFC not only impacts on power and performance but also on the specific role that the MFC will play (Rahimnejad et al., 2015; Rozendal et al., 2008). Focusing on single chamber MFCs, membraneless or membrane-based air-breathing cathode configurations are most often adopted. Research showed that membraneless MFCs produce higher current/power compared to membrane based configurations (Liu and Logan, 2004) however, fouling lowers system durability (Santini et al., 2017; Zhang et al., 2014), due to the cathode being directly exposed to wastewater. Whilst the addition of a membrane/separator can increase the ohmic losses (between anode and cathode) it plays an important role in electro-osmosis and separation between anode and cathode. This makes the system more durable and by operating as a barrier, oxygen is prevented from crossing over into the anode chamber and negatively affecting electrical output (Gajda et al., 2018; Salar Garcia et al., 2019).

To date, several separators have been employed with polymeric membranes being the material of choice (Das et al., 2018). Porous materials such as ceramics have received increased attention, as they are less likely to suffer from fouling (Behera et al., 2010). Another porous material is paper and considerable interest is being directed towards paper based MFCs because they are inexpensive, disposable and biodegradable (Winfield et al., 2015).

MFCs are capable of degrading a vast number of organics from simple molecules to more complex and diverse wastewaters (Aelterman et al., 2006; Pant et al., 2010; Pandey et al., 2016). Among these, human urine is a good feedstock because it contains a high concentration of organics and nutrients (Ieropoulos et al., 2012). Moreover, human urine possesses high solution conductivity, which is beneficial for lowering ohmic resistance and increasing power output. Urine has been used successfully as fuel for MFCs in several applications and field trials (Ieropoulos et al., 2016; Walter et al., 2018).

Considering that MFCs generate low power, a number of strategies need to be addressed to ensure that the electrical output can be used in real world applications (Ieropoulos et al., 2003; Wang et al., 2015). As already mentioned, these strategies include improvement of component materials and optimising reactor configuration as well as operational parameters; all this needs to be pursued with cost in mind. The current study addresses all these areas by developing paper-based MFCs and assessing the capacitive features of their electrodes in order to determine whether they can deliver high pulses of power compared to the lower power real-time output.

Researchers are realising that low cost, natural materials such as paper can be a viable replacement to conventional materials used in MFCs. Such materials will not be suitable for long term, large scale operation, such as wastewater treatment, but they can be suitable for special, short-term applications, e.g. diagnostics (Fraiwan et al., 2013), biosensors (Fraiwan et al., 2014), biodegradable robots (Rossiter et al., 2016), wearable electronics (Taghavi et al., 2015) and emergency power supplies in remote locations (Winfield et al., 2015) where an ‘expiry date’ is desirable.

For the technology to be truly considered sustainable, there needs to be a departure from using non-sustainable materials especially if the goal is a biodegradable or disposable product. Ferricyanide is still used in some cases for paper-MFCs (Nguyen and Taguchi, 2019) however this needs to be eradicated with alternative cathodic materials already identified e.g. graphite pencil drawn cathode (Lee et al., 2016). Another focus across a number of research groups has been stacking paper MFCs using kirigami and/or origami techniques. Through folding it is possible to introduce the relevant electrical connections into the system configuration e.g. folding in such a way that all MFCs are serially connected (Fraiwan and Choi, 2016). Other novel techniques include interconnecting individual paper MFC units in different ways to generate distinct configurations and characteristics (Fraiwan et al., 2016). These are innovative approaches but a challenge for paper-based MFCs is that their lifetime and therefore power supply is relatively short lived, sometimes only lasting minutes before the MFC undergoes a permanent decline. Three-dimensional paper-based pyramids have longer operational life and can generate stable outputs for months (Winfield et al., 2015), even passively feeding off the surrounding environment (Winfield et al., 2018). However, their individual real-time electrical output is not sufficient to directly power applications. One method for improving MFC performance is to operate them intermittently in order to generate bursts of energy (Ieropoulos et al., 2005; Walter et al., 2014). This is achieved because the energy accumulates across the MFC electrodes as they are also acting as supercapacitor electrodes (Deeke et al., 2013; Borsje et al., 2016; Santoro et al., 2016, Santoro et al., 2019a, Santoro et al., 2019b; Houghton et al., 2017). Therefore, galvanostatic discharges are applied over the internal supercapacitor resulting in high current/power in a short amount of time. This operating mode can be useful for powering discontinuously instruments that require high current/power intermittently.

To date, the supercapacitive MFC has only been tested using conventional MFC materials (Santoro et al., 2018b, Santoro et al., 2019a). In the current study, supercapacitor paper-based MFCs are reported for the first time. In the study, the anode and cathode of paper pyramid MFCs were considered as the two electrodes of an internal self-charged supercapacitor where galvanostatic discharges and different current pulses were analysed. Parameters of interest such as equivalent series resistance (ESR) and apparent capacitance (C) were identified and the relative contribution given by the single electrode is discussed. The stability of the system was investigated through 1000 discharge/self-recharge cycles in which ESR and C were measured over time.

## 2 Materials and methods

### 2.1 Microbial fuel cell fabrication

**Fig. 1:**
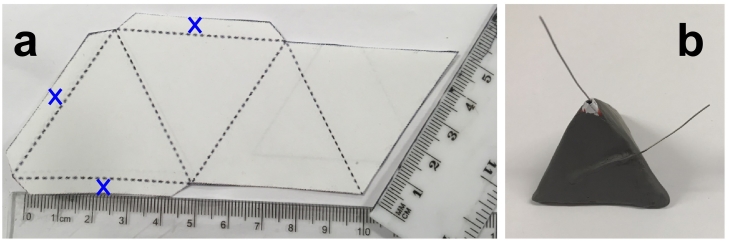
Pyramid MFC template prior to folding (a). The dotted line indicates where the paper is folded. Image of the pyramid MFC (b). (For interpretation of the references to colour in this figure, the reader is referred to the web version of this article.)

For this experimental study, two identical MFCs, named as MFC-A and MFC-B, were fabricated using Image® 100% recycled white paper with a weight of 80 g m^−2^ (Antalis™, France). The MFC shape was cut out of the aforementioned paper using the template shown below (Fig. 1). The anode electrode was fabricated out of untreated carbon veil (10 g m^−2^, Optiveil® 20301A, Technical Fibre Products, UK) with a total geometric area of 81 cm^2^. It was folded down into a triangular shape to fit inside the pyramid whilst allowing empty space for the liquid to percolate through. As a current collector, a piece of stainless steel wire was threaded through the carbon veil, holding it together. The anode was held inside the paper shape as it was folded, then the edges (blue cross highlighted in Fig. 1a) were glued together using Bostik glue (All Purpose Clear Adhesive) to create a pyramid shape that acts as a permeable membrane between anode and cathode (with the anode inside). On the outside, the pyramid was painted with three layers of conductive latex (Plasti-dip) as previously described (Winfield et al., 2015) that formed the air exposed cathode. As a cathodic current collector, a stainless steel wire was attached with wire glue on the paper before applying the three layers of cathode. Conductive latex sealed the three upper sides of the structure and as well as functioning as the cathode, this also prevented liquid from escaping through the paper. The bottom of the pyramid was coated with a layer of Plasti-dip (non-conductive) to eliminate leakages. The entire structure was able to hold 15 mL.

### 2.2 Inoculum and operation

The identical MFC-A and MFC-B, were fed using a syringe through a small incision in the top of the pyramid. To inoculate, anolyte was taken from the anode chambers of separate, established MFCs, and mixed 50:50 with fresh urine. This was fed to the MFCs and topped up every day for three days. Due to the MFC architecture and material used, there was never a need to remove anolyte because the MFCs naturally lost up to half of their volume through evaporation every 24 h. Following the three days of inoculation, 100% fresh urine was supplied to the cells daily until the end of the experiment. Urine was collected from healthy individuals at the Bristol Robotics Laboratory, Bristol, UK. The MFCs were operated under an external resistor of 1 kΩ which was applied once the open circuit voltage of the MFCs reached a plateau (within 3 hours from inoculation). The experiment was performed under identical experimental conditions and at ambient temperature (22 ± 1 °C).

### 2.3 Electrochemical measurements

The two identical paper based MFCs, named as MFC-A and MFC-B, were connected to an external resistance and after a week, were left in open circuit voltage (OCV) for at least 2 h before discharging. Biologic SP-50 potentiostat was used for performing galvanostatic discharges (GLV). The MFC was in galvanostatic mode and different current pulses (**i**_**pulse**_) and pulse lengths (**t**_**pulse**_) were selected. GLVs were performed utilising a three electrode configuration in which Ag/AgCl 3 M KCl (Φ 2 mm) was used as the reference electrode, the cathode as the working electrode and the anode as the counter electrode. The reference electrode was inserted through the hole in the top of the pyramid and immersed into the electrolyte.

The MFC was left in OCV without being connected to an external resistance. This is termed ‘*rest condition*’ and the measured voltage is named as **V**_**max**,**OC**_, which is the difference in potential between the anode and cathode. As the two red-ox reactions were occurring, the anode was charged negatively while the cathode was charged positively. Full discharges (from **V**_**max**,**OC**_ to 0 mV) were carried out using different **i**_**pulse**_. After a discharge, the voltage returned back to **V**_**max**,**OC**_, and the electrodes would self-recharge again.

During discharging, the voltage varied and particularly, as the **i**_**pulse**_ starts, a vertical drop can be noted. This drop is named as **ΔV**_**ohmic**_. From **ΔV**_**ohmic**_, it is possible to determine the ohmic losses of the MFC. These losses are named as Equivalent Series Resistance (**ESR**), which includes the ohmic resistance of the anode, the cathode and the electrolyte. **ESR** can be calculated as shown in Eq. (1) below.

(1) $$\mathrm{ESR}=\frac{∆V_{\mathrm{ohmic}}}{i_{\mathrm{pulse}}}$$

The utilisation of the reference electrode enables the contribution of the two electrodes to be separately characterised. Because the reference electrode was close to the anode electrode, the ohmic losses are caused specifically by the ohmic resistance of the anode material (**R**_**A**_). The ohmic losses of the cathode electrode (**R**_**C**_) on the other hand were not only a result of the ohmic resistance of the material but also caused by the resistances of the electrolyte (the reference electrode was approximately 3 cm away from the cathode) and the paper separator. The sum of **R**_**A**_ and **R**_**C**_ gives the **ESR** according to Eq. (2).

(2) $$\mathrm{ESR}=R_{A}+R_{C}$$

Following the drop, a new voltage point (**V**_**max**_) was reached. After this point, the MFC voltage keeps decreasing until it reaches 0 mV (full discharge). Another important parameter to consider is the capacitance. The apparent capacitance is calculated according to Eq. (3).

(3) $$C_{\mathrm{MFC}}=\frac{i_{\mathrm{pulse}}}{\frac{{\mathrm{dV}}_{\mathrm{tot}}}{\mathrm{dt}}}=\frac{i_{\mathrm{pulse}}}{s}$$

The apparent capacitance is the ratio between the i_pulse_ and the variation in voltage from V_max_ to 0 mV. The voltage variation from V_max_ to 0 mV is also the slope of the curve (named s). The expression “apparent” is used to describe the environment, where the electrochemical double layer (EDL) is formed and faradaic reactions are taking place during discharge. The utilisation of the reference electrode enables the evaluation of the apparent capacitance of both anode and cathode according to Eqs. (4) and (5):

(4) $$C_{A}=\frac{i_{\mathrm{pulse}}}{\frac{{\mathrm{dV}}_{A}}{\mathrm{dt}}}$$

(5) $$C_{C}=\frac{i_{\mathrm{pulse}}}{\frac{{\mathrm{dV}}_{C}}{\mathrm{dt}}}$$

C_MFC_, C_A_ and C_C_ are related by Eq. (6) as detailed below:

(6) $$C_{\mathrm{MFC}}={\left(\frac{1}{C_{A}}+\frac{1}{C_{C}}\right)}^{-1}$$

During galvanostatic discharges, power and energy are also evaluated. The energy delivered during the pulse (E_pulse_) is calculated according to Eq. (7):

(7) $$E_{\mathrm{pulse}}=i_{\mathrm{pulse}}\int_{0}^{t}V\;\mathrm{dt}$$

where i_pulse_ is the pulse current and the integral is the area below the voltage profile during the discharge.

E_pulse_ and P_pulse_ are related with the t_pulse_ according to Eq. (8):

(8) $$P_{\mathrm{pulse}}=\frac{E_{\mathrm{pulse}}}{t_{\mathrm{pulse}}}$$

In this work, a range of different **t**_**pulses**_ was investigated (0.01 s, 0.05 s, 0.1 s, 0.5 s, 1 s, 2 s and 5 s).

## 3 Results and discussion

### 3.1 Full discharge analysis

**Fig. 2:**
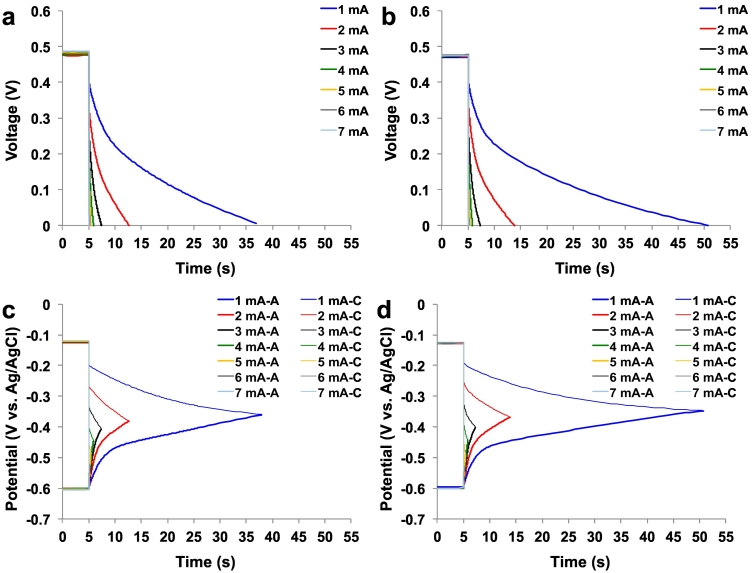
Complete discharges of the paper based MFC. Overall profiles for MFC-A (a) and MFC-B (b). Single electrode profile for MFC-A (c) and MFC-B (d).

Complete discharges with i_pulse_ varying from 1 mA to 7 mA were performed (Fig. 2) with MFC-A presented in Fig. 2a, and MFC-B discharges shown in Fig. 2b. The separate anode and cathode trends for MFC-A and MFC-B are presented in Fig. 2c and d, respectively. Firstly, it can be noted that the **V**_**max**,**OC**_ for MFC-A was 481 ± 4 mV which is 8 mV higher than the OCV of MFC-B (473 ± 2 mV).

The anodic potential levels of MFC-A and MFC-B were similar (−604 ± 2 mV and −600 ± 2 mV vs Ag/AgCl respectively) and were comparable to the expected theoretical value based on the oxidation of NADH/NAD^+^ (−630 mV at pH 9 vs Ag/AgCl) (Logan et al., 2006). Similar values were also found previously in self-stratified supercapacitive MFCs operating with urine (Santoro et al., 2019b).

The cathode potential levels were −122 ± 3 mV vs Ag/AgCl (MFC-A) and −127 ± 1 mV vs Ag/AgCl (MFC-B). The reduction of oxygen occurring at pH = 9 has a redox potential of ≈+487 mV vs Ag/AgCl considering a 4e- transfer mechanism. This indicates that the cathode catalyst adopted for this investigation was extremely poor and the activation overpotentials were roughly 600 mV. In self-stratified supercapacitive MFCs operating with urine presented previously (Santoro et al., 2019b), cathode potential was ≈+150 mV vs Ag/AgCl. This indicates were poor catalytic activity of the cathode utilised in this experimentation.

ESR was 44.5 ± 2.3 Ω for MFC-A and 38.2 ± 1.1 Ω for MFC-B. For MFC-A, R_A_ and R_C_ were also evaluated and were 2.2 ± 0.2 Ω and 42.2 ± 2.2 Ω respectively. MFC-B had similar R_A_ to MFC-A (1.0 ± 0.3 Ω) but a lower R_C_ (36.7 ± 1.0 Ω). As mentioned before, R_A_ could be considered as the ohmic resistance of the material while the R_C_ contains the contribution of electrolyte, separator and cathode. Indeed, R_C_ contributed 95% of the total ESR. Higher R_C_ compared to R_A_ is in agreement with previously reported work on supercapacitive MFCs (Houghton et al., 2016; Santoro et al., 2017).

The apparent capacitance (CMFC) was dependent on the t_pulse_, which increased as the t_pulse_ became shorter. With respect to the current pulses, the apparent capacitance dropped as the current dropped; i.e. from 7 mA to 1 mA the apparent C_MFC-A_ was: 76.0 mF, 40.1 mF, 21.2 mF, 12.3 mF, 8.2 mF, 4.7 mF and 2.5 mF.

This phenomenon was similar for MFC-B with the apparent capacitance values being 106.6 mF, 45.0 mF, 19.7 mF, 10.5 mF, 6.5 mF, 3.4 mF and 0.9 mF for decreasing current pulses from 7 mA to 1 mA. The anode and cathode apparent capacitance for MFC-A and MFC-B are reported in Table 1. Cathode apparent capacitance (except for MFC-B at 1 mA i_pulse_) is generally higher than the anode apparent capacitance indicating the poor capacitive features of carbon veil in agreement with Santoro et al. (2019b).

**Table 1:** C_tot_, C_A_ and C_C_ values for MFC-A and MFC-B.

MFC-A				MFC-B		
i_pulse_	C_tot_	C_A_	C_C_	C_tot_	C_A_	C_C_
mA	mF	mF	mF	mF	mF	mF
1	76.02	133.60	168.05	106.57	346.36	249.84
2	40.14	71.18	92.06	44.95	77.88	106.96
3	21.17	37.91	47.95	19.69	36.03	43.42
4	12.33	24.50	24.83	10.51	22.35	19.83
5	8.15	19.85	13.83	6.47	17.81	10.16
6	4.67	17.13	6.41	3.43	15.00	4.44
7	2.48	16.66	2.91	0.92	14.00	0.97

**Fig. 3:**
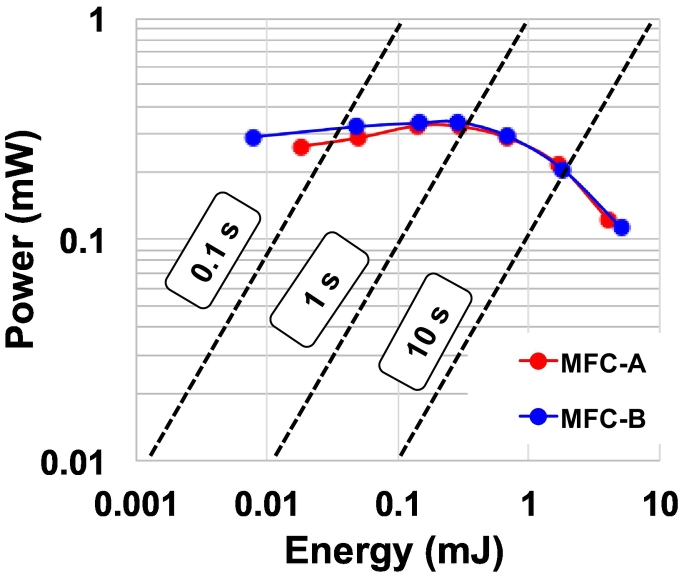
Ragone's plot for MFC-A and MFC-B.

When the i_pulse_ was lower, the discharge time was longer. For example, the t_pulse_ for complete discharge (MFC-A) was 31.93 s (1 mA), 7.69 s (2 mA), 2.41 s (3 mA), 0.91 s (4 mA), 0.43 s (5 mA), 0.17 s (6 mA) and 0.07 s (7 mA). The pattern was similar for MFC-B where the complete discharges occurred in 45.72 s (1 mA), 8.88 s (2 mA), 2.33 s (3 mA), 0.84 s (4 mA), 0.37 s (5 mA), 0.15 s (6 mA) and 0.03 s (7 mA). The comparison in performance between the two MFCs during the complete discharge cycles is illustrated in the Ragone plot (Energy vs Power) (Fig. 3).

### 3.2 Maximum power curves and pulsed power curves

**Fig. 4:**
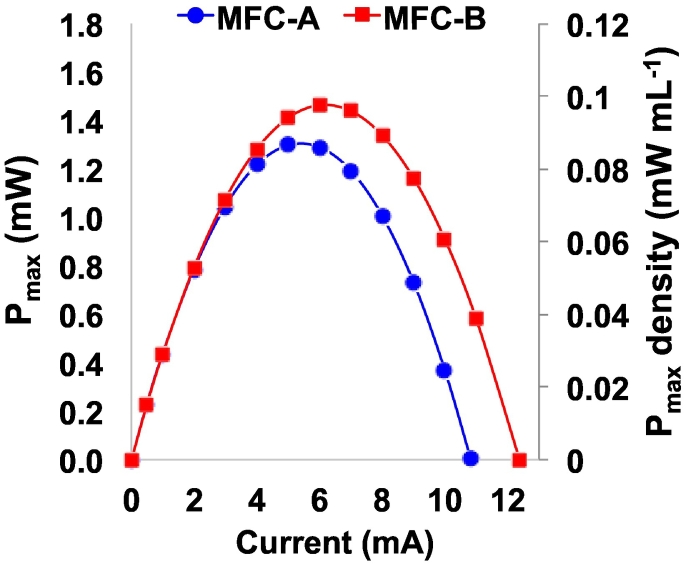
Calculated P_max_ curves for MFC-A (blue) and MFC-B (red). (For interpretation of the references to colour in this figure legend, the reader is referred to the web version of this article.)

It was possible to calculate the P_max_ (maximum achievable power) when the V_max,OC_ and the ESR are known. These values were 482 mV (vs Ag/AgCl) and 44.5 Ω for MFC-A and 473 mV (vs Ag/AgCl) and 38.2 Ω for MFC-B. The resulting P_max_ curves are shown in Fig. 4. MFC-A and MFC-B had similar s V_max,OC_ and ESR which is reflected in their comparable P_max_ values measuring 1.298 mW (0.087 mW mL^−1^) and 1.463 mW (0.098 mW mL^−1^) respectively.

**Fig. 5:**
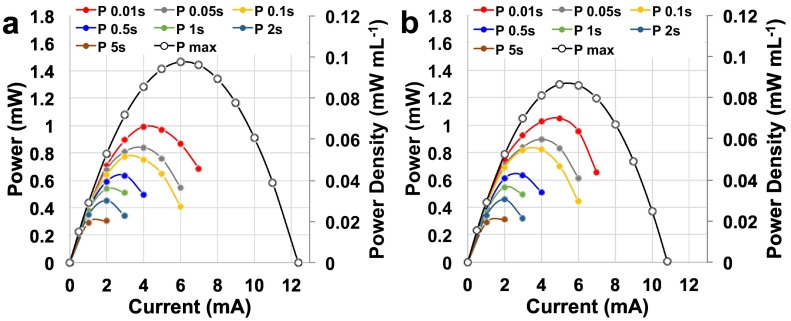
P_pulse_ for MFC-A (a) and MFC-B (b) for t_pulse_ of 0.01 s, 0.05 s, 0.1 s, 0.5 s, 1 s, 2 s and 5 s.

P_pulse_ for t_pulse_ of 0.01 s, 0.05 s, 0.1 s, 0.5 s, 1 s, 2 s and 5 s were calculated for MFC-A (Fig. 5a) and MFC-B (Fig. 5b). The shortest pulse was the GLV pulse, while the higher one was the P_pulse_ (Fig. 5). Therefore, the shorter t_pulse_ investigated (0.01 s) gave the highest outputs (0.992 mW (0.066 mW mL^−1^) for MFC-A and 1.048 mW (0.069 mW mL^−1^) for MFC-B). At t_pulse_ of 0.05 s, P_pulse_ was 0.840 mW (0.056 mW mL^−1^) and 0.896 mW (0.059 mW mL^−1^) for MFC-A and MFC-B. P_pulse_ for t_pulse_ 0.1 s was recorded as 0.774 mW (0.052 mW mL^−1^) and 0.820 mW (0.055 mW mL^−1^). At t_pulse_ of 0.5 s, the P_pulse_ was 0.636 mW (0.042 mW mL^−1^) for both MFCs. Lower P_pulse_ were recorded for t_pulse_ of 1 s (0.538 mW (0.036 mW mL^−1^) and 0.548 mW (0.037 mW mL^−1^)). The longer pulses gave the lowest P_pulse_ values (Fig. 4). At 2 s t_pulse_, the P_pulse_ produced was 0.455 ± 0.002 mW equivalent to a volumetric power density of 0.030 mW mL^−1^. At the longest t_pulse_ investigated, the P_pulse_ was 0.304 mW (0.020 mW mL^−1^) and 0.315 mW (0.021 mW mL^−1^). Interestingly, the difference between the two MFCs was always below 7% indicating consistency in the fabrication process and electrochemical evaluation techniques. Compared to the self-stratifying supercapacitive MFCs, the volumetric power is lower probably due to the lower operating voltage as well as the not optimised electrode area to volume ratio of the paper based MFC.

### 3.3 Durability tests: analysis over 1000 cycles

**Fig. 6:**
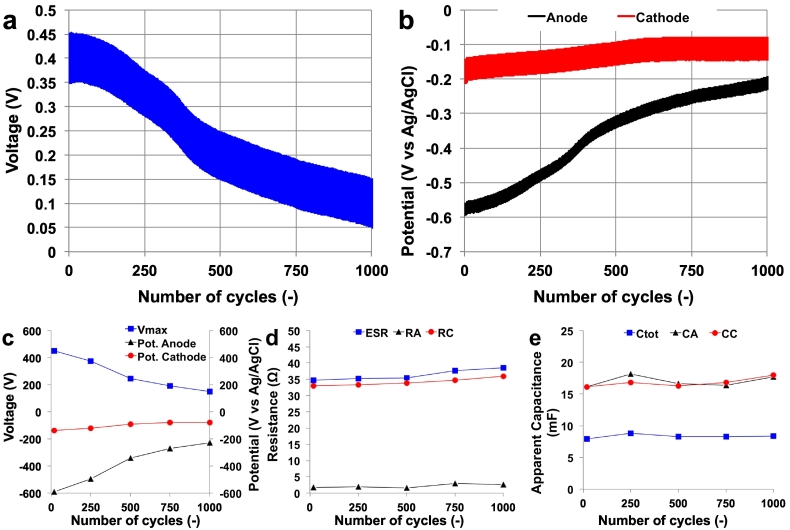
1000 cycles discharges/self-recharging. Overall (a) and single electrode trend (b). Voltage and potential variation over time (c). Variation of ESR, R_A_ and R_C_ (d) and apparent C_tot_, C_A_ and C_C_ (e) over the cycles.

Long-term discharge/self-recharge cycles were run over ≈24 h using an i_pulse_ of 1 mA and a t_pulse_ of 0.5 s. Preliminary results showed that 85 s was necessary to recover the initial voltage/potential values. 1000 cycles of discharge/self-recharge are presented in Fig. 6. The overall trend (Fig. 6a) and the anode and cathode trends (Fig. 6b) were evaluated over 24 h. At the beginning, the MFC had a V_max,OC_ of 450 mV with anode and cathode potentials being −590 mV vs Ag/AgCl and −139 mV vs Ag/AgCl respectively. There was a slight increase in the cathode potential over time but a significant decrease in the anode potential. The latter had a negative affect on the overall cell potential. Particularly, the cathode OCP was −119 mV vs Ag/AgCl after 250 cycles, −93 mV vs Ag/AgCl after 500 cycles, −79 mV vs Ag/AgCl after 750 cycles and −79 mV vs Ag/AgCl at the end of the experiment (1000 cycles) (Fig. 6c). This increase might be due to the evaporation of liquid from the MFC with the consequent increase in salt concentration (especially chlorine (Lammel et al., 2017)) that can shift the reference potential.

Concerning the anode OCP, the value measured was −494 mV vs Ag/AgCl after 250 cycles, −340 mV vs Ag/AgCl after 500 cycles, −270 mV vs Ag/AgCl after 750 cycles and −228 mV vs Ag/AgCl at the end of the experiment (1000 cycles). Therefore, the cell potential diminished from 450 mV (0 cycles) to 374 mV (250 cycles) and 246 mV (500 cycles) dropping to 149 mV by the end of the experiment (Fig. 6c). This decrease might be due to two reasons: i) rapid consumption of easily degradable organics in urine occurring in the first few hours after refilling; ii) evaporation of urine over time. The latter is the most probable cause since after 24 h, 6.5 mL out of 15 mL (43%) had evaporated from the MFC. This evaporation might have exposed the anode to air and/or decreased the active surface interface between anode and cathode i.e. the top half of the anode was not submerged and so was not reacting with the upper cathodic layer on the other side of the paper separator.

The ESR, R_A_, R_C_, C_MFC_, C_A_ and C_C_ were also evaluated during the operation. ESR slightly increased during operation rising from 34.7 Ω (0 cycles) to 38.6 Ω (1000 cycles) (Fig. 6d). This increase was mainly due to the variation of R_C_ that increased from 33.0 Ω (0 cycles) to 36.0 Ω (1000 cycles) (Fig. 6d). R_A_ was fairly stable, increasing slowly from 1.7 Ω and 2.9 Ω (Fig. 6d).

There was a slight increase in apparent capacitance (C_tot_) but overall it remained fairly constant during the experiments rising from 7.90 mF (cycle 0) to 8.36 mF (cycle 1000) (Fig. 6e). Apparent C_A_ also increased slightly during the experimentation, measuring 16.13 mF (cycle 0) to 17.73 mF (cycle 1000) (Fig. 6e). A small improvement in apparent C_C_ was also detected (Fig. 6e). Interestingly, the anode and the cathode apparent capacitance had similar contributions to the overall apparent capacitance of the MFC.

Despite a decrease in performance over the 1000 cycles, the resistance (ESR, R_A_, R_C_) and the apparent capacitance (C_tot_, C_A_, C_C_) remained stable or varied only slightly during operation. The variation in the anode potential was the main cause for the decrease in electrochemical output.

### 3.4 Outlook

Using conventional MFCs, it has been previously shown that galvanostatic discharges produce higher electrochemical output compared to continuous operational mode (Santoro et al., 2016, Santoro et al., 2017, Santoro et al., 2019a, Santoro et al., 2019b). This is the first time that a paper based MFC has been used to investigate the electrode behaviour when operating as a supercapacitor. Paper separators have the advantage of being inexpensive and lightweight and with a respectable power output - the maximum power (P_max_) recorded was 1.380 ± 0.083 mW (0.092 ± 0.006 mW mL^−1^). The anode electrochemical potential was similar to the theoretical one whereas the cathode OCP was roughly 500 mV lower than the expected theoretical value. This indicates that the cathode and the catalyst used were suboptimal for the role, but they did contribute to a simple and low cost design. The power recorded in this work was greater compared to the one achieved by Winfield et al. (2015) operating in continuous mode, which was ≈0.055 mW (≈0.0037 mW mL^−1^). In order to power practical applications, a number of supercapacitive MFCs should be connected in series and parallel in order to boost up voltage and current; this will be part of a future investigation. Future work will also investigate other cathode materials particularly with a focus on inexpensive biodegradable alternatives. Also the cathode fabricated using the conductive latex did not give high apparent capacitance. These considerations give rise to the possibility of further improvements of the MFCs through materials design and development with the intention of boosting even further power/energy output. Further investigations will also address optimising the cathode side in order to increase the electrode potential and enhance the operating voltage window. Moreover, capacitive features have to be included in the electrodes for increasing electrode capacitance and therefore the power/energy delivered. Another aspect to be considered, is the reduction in liquid level, due to evaporation that negatively affects the anode performance and the overall cell output. Strategies for decreasing the liquid evaporation need to be adopted for enhancing durability. Particularly, the next suite of experiments will focus on a range of waterproofing materials including plasti-dip, lanolin and natural rubber, as cathode coatings. Preliminary experiments showed that coatings such as these significantly limit evaporation. This study has used paper-based MFCs to illustrate that higher bursts of power can be generated by tapping into the capacitive nature of MFC electrodes. Future work will focus on building supercapacitive paper-based MFCs capable of energising real world systems.

## 4 Conclusions

Paper based MFCs were tested in supercapacitive mode. Complete galvanostatic discharges were performed and ESR was evaluated (41.2 ± 3.5 Ω) with the cathode contributing to over 94% of losses. Apparent capacitance increased under lower current pulses. A maximum power output of 1.380 ± 0.083 mW (0.092 ± 0.006 mWmL^−1^) was achieved by duplicate paper based MFCs however lower power output was recorded for longer pulses. 1000-cycle (24 h) durability tests were conducted. Resistance and apparent capacitance did not vary significantly, however, the anode potential did increase probably due to the evaporation of liquid inside the MFC exposing the anode to aerobic environments.
